# Dynamic assessment of proliferation to guide response-adapted therapy in the setting of neoadjuvant chemotherapy in ER+/HER2- breast cancer

**DOI:** 10.1016/j.tranon.2025.102597

**Published:** 2025-11-15

**Authors:** Hani Saghir, Srinivas Veerla, Niklas Loman, Siker Kimbung

**Affiliations:** a Division of Oncology, Department of Clinical Sciences, Lund University, Lund, Sweden; b Lund University Cancer Center, Lund, Sweden; c Department of Hematology, Sahlgrenska University Hospital, Gothenburg, Sweden; d Department of Hematology, Oncology, and Radiation Physics, Lund University Hospital, Malmö/Lund, Sweden

**Keywords:** Biomarkers, Proliferation, Gene expression assays, ER+/HER2- Breast Cancer

## Abstract

Biomarkers for evaluating response to neoadjuvant chemotherapy (NACT) in ER+/HER2− breast cancer remain limited. This study explores the impact of NACT on tumor proliferation dynamics and its association with relapse-free interval (RFI) among 175 patients with early ER+/HER2− breast tumors. Proliferation was assessed at baseline, after completing two NACT cycles, and in the residual tumor using Ki67 immunohistochemistry (IHC) and two gene expression assays (GEAs): SSP-Ki67 and AURKA score. Slight to moderate agreement was observed between IHC and GEAs, with IHC-Ki67 consistently classifying more tumors as highly proliferative compared to SSP-Ki67 at both baseline and surgery. Proliferation status at baseline was not prognostic for RFI using either IHC-Ki67 or SSP-Ki67 in our cohort. However, patients with persistently high proliferation after two NACT cycles or in the residual tumor following NACT were at increased risk of relapse, with SSP-Ki67 outperforming IHC-Ki67 in identifying patients with a poorer prognosis. Our results demonstrate that tumor proliferation status measured after brief exposure to NACT or in the residual tumor post-NACT holds prognostic value and may inform the tailoring of post-neoadjuvant treatment strategies in patients with early luminal breast cancer, and that relying on IHC-Ki67 to evaluate treatment response may potentially lead to overtreatment.

## Highlights

• GEAs accurately assess proliferation in ER+/HER2- breast cancer.
• GEAs classify fewer patients as highly proliferative compared to IHC.
• Treatment-induced changes in proliferation can identify patients at high relapse risk.
• GEA-guided response assessment may support treatment de-escalation trials.

## Introduction

Estrogen receptor-positive (ER+)/ Human epidermal growth factor receptor 2 negative (HER2-) breast cancer is the most common subtype of breast cancer, accounting for approximately 80 % of primary cases. While it has a better prognosis than other subtypes, relapse is common [1,2]. Risk stratification in ER+/HER2- breast cancer is crucial for optimal treatment selection. It incorporates clinical risk assessment based on the biomarkers estrogen receptor (ER), progesterone receptor (PgR), Ki67, HER2, Nottingham histological grade (NHG), and clinical staging [2].

Gene expression assays (GEAs) assess the genomic recurrence risk of the tumor, usually identifying patients with a low genomic risk for disease recurrence despite having a high clinical risk for recurrence, allowing safe omission of chemotherapy. Prospective trials and retrospective analyses support this approach with assays like OncotypeDX, Mammaprint, and PAM50 Risk of Recurrence (ROR) approved for use in decisions concerning chemotherapy treatment in the adjuvant setting [[3], [4], [5], [6], [7]].While these gene expression signatures [[3], [4], [5], [6], [7]] are established tools for guiding adjuvant therapy in ER+/HER2– early breast cancer, their role in predicting response to neoadjuvant treatments remains exploratory with limited evidence as outlined in a review from 2025 [8]. Most retrospective studies suggest that non-luminal intrinsic subtypes and higher genomic risk scores are associated with higher rates of pathological complete response (pCR) [9].

Preoperative treatments like NACT and neoadjuvant endocrine therapy (NET) offer a unique opportunity to evaluate tumor biological response in vivo, enabling response-adapted therapy. However, the choice between NACT and NET in ER+/HER2- early breast cancer is still based on clinical and pathological features and is not guided by genomic biomarkers of defined clinical utility.

Pathological complete response (pCR) is a well-established surrogate endpoint for evaluating response to NACT and prediction of long-term clinical benefit of NACT [10]. While pCR is strongly prognostic in triple-negative breast cancer (TNBC) and HER2+ disease, its prognostic value in ER+/HER2- disease is limited since such patients rarely respond with a pCR.

Response-adapted therapy improves outcomes in TNBC and HER2+ breast cancer. In patients with TNBC who have residual disease after NACT, adjuvant capecitabine (as demonstrated in the CREATE-X study) and Olaparib for BRCA-associated cases (as shown in the OlympiA study) significantly extend disease-free survival. Similarly, in patients with HER2+ tumors, the adjuvant antibody-drug conjugate trastuzumab-emtansine enhances outcomes [[11], [12], [13]].

Identifying novel biomarkers for optimal response prediction and evaluation to preoperative treatment in ER+/HER2- disease could enable more precise treatment escalation or de-escalation. Also, patient safety is improved by avoiding non-beneficial yet toxic therapies. While NACT is a less common treatment choice in early ER+/HER2- breast cancer, identifying better biomarkers to optimally predict long-term response could make NACT a more viable option, enabling response-adapted treatment allocation during the neoadjuvant or adjuvant treatment phases and facilitating the identification of high-risk patients and patients with predicted favorable outcomes.

New approaches, such as dynamic immunohistochemical assessment of the proliferation biomarker Ki67 (IHC-Ki67), show promise in refining prognosis and treatment strategies. For example, in the ADAPT study, interim IHC-Ki67 analysis after 3 weeks of NET treatment enabled safe de-escalation of adjuvant chemotherapy [14]. In the POETIC study, low IHC-Ki67 after 2 weeks of NET predicted favorable outcomes, with over 50 % reduction in the risk for recurrences compared to persistently high IHC-Ki67 levels [[15], [16]]. While the prospect of using IHC-Ki67 expression to evaluate tumor biological response seems promising, IHC-Ki67 is infamous for its analytical validity. The International Ki67 Working Group (IKWG) recommends limiting its clinical utility and highlights many of its limitations in a report from 2019 [17].

Further highlighting the limited analytical validity of IHC-Ki67, our previous study comparing IHC-Ki67 classification between therapy-naïve core-needle biopsy (CNB) and paired surgical resection found a concordance rate of 82.9 % for high or low classification, with a disproportionate trend favoring switches from low expression in the CNB to high expression in the surgical specimen. While IHC-Ki67′s utility is limited by immunohistochemical staining issues, our previous work suggests that measuring proliferation using GEAs may provide a more accurate assessment of tumor proliferation status [18].

The primary aim of this study was to investigate how NACT-induced changes in tumor proliferation impact long-term outcomes among patients with ER+/HER2- early breast cancer. Specifically, we assess the prognostic significance of proliferation status derived from RNAseq data using a single sample predictor gene signature for Ki67 (SSP-Ki67), developed by Sweden Cancerome Analysis Network-Breast (SCAN-B) [19] in comparison with Ki67 status determined by conventional immunohistochemistry (IHC-Ki67). Additionally, we explore correlations between the IHC-Ki67 and SSP-Ki67 classifications with an independent proliferation gene signature, AURKA proliferation score (AS), proposed by Desmedt et al. [20]. Finally, treatment-induced changes in tumor proliferation are compared to objective tumor size reduction as a secondary objective.

We hypothesize that NACT-induced reduction in tumor proliferation is prognostic for better outcome and that GEAs can overcome IHC-related technical and analytical challenges in assessing tumor proliferation status, providing a more precise and reproducible assessment of tumor proliferation to guide response-adapted therapy in ER+/HER2- breast cancer.

## Materials and methods

### Study population and sample collection

This study is based on the Sweden Cancerome Analysis Network-Breast (SCAN-B) cohort, an ongoing multicenter observational study launched in September 2010 to analyze breast tumors using next-generation genomics for translational research. As of January 2025, SCAN-B has enrolled 22,013 patients. Refer to [clinicaltrials.gov/study/NCT02306096] and the SCAN-B website (https://www.scan-b.lu.se/) for the complete inclusion and exclusion criteria. All patients provided written informed consent before enrollment into SCAN-B. The study, including translational projects, was approved by the Regional Ethical Review Board of Lund (registration numbers 2009/658, 2010/383, 2012/58, 2013/459 2014/521, 2015/277, 2016/944), the county biobank center, and the Swedish Authority for Privacy Protection (registration number 364–2010). Sample collection and processing within SCAN-B has been previously described [21,22].

This sub-study included *n* = 175 women with ER+/HER2- stage I-III breast cancer treated with NACT between 2010 and 2019. No formal power calculation was performed prior to study initiation; instead, all available tumor samples meeting the inclusion criteria were included in this exploratory analysis. At least two separate pre-NACT CNBs were obtained at baseline via ultrasound guidance for histopathology and RNA sequencing (RNAseq) respectively, as part of SCAN-B routines [21]. A second CNB (*n* = 39) was taken for interim RNA sequencing wherever possible with consent from the patient after completing two NACT cycles. After six NACT cycles, samples from the residual tumor were collected for histopathology and RNAseq analyses. Clinical data including disease recurrences were extracted directly from patient medical records.

**Fig. 1:**
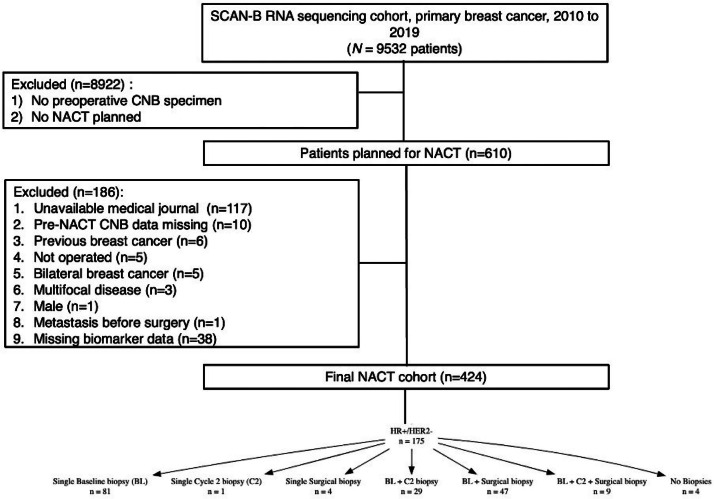
Flowchart illustrating the number of patients included in the study and the biopsies with good quality RNA sequencing data, including the combinations of biopsy timepoints analyzed.

Details on the selection of patients and tumors for this sub-study are presented in Fig. 1. Cases with bilateral disease, multifocal disease, radiologically occult breast cancer, previous breast cancer, or no successful IHC biomarker status on the pre-NACT CNB were all excluded. The numbers of patients with good-quality RNAseq data at sequential time points during treatment are present in Fig. 1.

### Immunohistochemical analysis

Local pathology laboratories performed IHC analyses of all conventional biomarkers following the detailed protocols of the Swedish Society of Pathology’s Quality and Standardization Committee (KVAST) [23] applicable at the time of diagnosis. Tumor tissue was fixed in 10 % neutral buffered formalin for 24–72 h, with a cold ischemia time of less than one hour. Formalin-fixed, paraffin-embedded (FFPE) sections were stained using immunohistochemistry and performed using clinically validated antibodies: Estrogen receptor (ER, clone SP1, prediluted, Ventana, Ref. 790–4324), Progesterone receptor (PgR, clone 1E2, prediluted, Ventana, Ref. 790–2223), Ki-67 (clone MIB-1, 1:50 dilution, Agilent, Ref. M724001–2), and HER2 (clone 4B5, prediluted, Ventana, Ref. 790–4493). Appropriate positive and negative controls were included to validate antibody performance. Biomarker status annotation was performed by board-certified pathologists. IHC-Ki67 index was determined by counting at least 200 cells in hotspot areas using either microscopy or digital image analysis, and results are reported as a continuous percentage score for positive nuclei fraction. ER and PgR positivity was defined as ≥10 % positive nuclei fraction, according to Swedish guidelines. HER2 status was classified as negative (IHC 0–1+), positive (IHC 3+), or for IHC 2+ cases, confirmed via FISH (Fluorescent in situ hybridization) or SISH (Silver-enhanced in situ hybridization) for HER2 amplification[23]. Nottingham histological grade (NHG) data was only available from the surgical specimens, in accordance with Swedish routine clinical practice at the time of patient inclusion.

### Gene expression assays (GEAs)

RNA sequencing of fresh tumors was conducted centrally at the SCAN-B laboratory [21]. Tumor handling procedures, RNA sequencing and data preprocessing in SCAN-B have been extensively described [19,21]. In brief, preoperative CNBs were obtained by radiologists under ultrasound guidance and immediately preserved in RNAlater reagent. Fresh tumor samples preserved in RNAlater were transported in real-time from all participating clinical sites to the SCAN-B central laboratory in Lund, Sweden, where RNA sequencing is performed directly, as previously described [19,21].

Nucleic acids are isolated using the Qiagen AllPrep kit. Quality control of nucleic acids is performed using NanoDrop spectrophotometry and capillary electrophoresis with the Agilent BioAnalyzer. RNA-sequencing was performed by Illumina stranded TruSeq mRNA protocol, either implemented on KingFisher or on the Illumina NeoPrep system. Expression data (Fragments Per Kilobase per Million reads, FPKM) from Stringtie was derived from RNA-sequencing data using a SCAN-B analysis pipeline, which is based on open-source software. Analysis steps are implemented as an automated analysis pipeline in BASE [24] with extension package Reggie (BioRxiv: https://doi.org/10.1101/038976). GRCh38 [25], dbSNP [26], and GENCODE [27] were used to create alignment and transcript targets.

To determine the tumor Ki67 status using gene expression data, a single sample predictor (SSP) gene signature model for predicting Ki67 status (SSP-Ki67) for individual tumors was implemented as described originally [19]. The SSP-Ki67 was trained on the respective IHC-Ki67 label (using local cut-offs to differentiate high from low proliferation) and showed moderate agreement with the IHC-Ki67 status in the test and validation cohorts (accuracy = 80 %, Kappa = 0.59), as described by Staaf et al. [19].

Finally, we calculated the corresponding AURKA score (AS) for each tumor sample using RNAseq data [20]. AS is a tumor proliferation signature measured on a continuous scale. Supplementary Table S1 from the referenced publication [20] was used to extract a list of 229 genes, along with their respective coefficients associated with the AURKA module score for tumor proliferation status. The corresponding AURKA score (AS) for each tumor sample in our cohort was calculated using RNAseq data strictly following the previous guidelines [20]. Specifically, the normalized FPKM value for each gene included in the module was multiplied by its corresponding coefficient, and the sum of the products yielded the final AS for the individual tumor sample.

### Cut-off values for IHC-Ki67

Local IHC cut-offs for stratifying Ki67 expression into low versus high ranged between 17 and 31 %, depending on the pathology laboratory, as specified by the Swedish Society of Pathology’s Quality and Standardization Committee (KVAST) at the time of the study [23]. According to these guidelines, tumors were categorized as low- or high-proliferative based on each laboratory’s cut-off, defined by the upper one-third of invasive tumors with the highest proliferation. In this study, however, a uniform cut-off of ≥10 % was applied to align with previous research on dynamic Ki67 assessment after NET [14,15], unless otherwise stated.

### Statistical analysis

To evaluate the association between proliferation dynamics and long-term outcome, we used the endpoint relapse-free interval (RFI) in which an ipsilateral breast cancer recurrence, a locoregional recurrence, distant metastasis, or death from breast cancer are included events according to the Standardized Definitions for Efficacy End Points (STEEP) criteria version 2.0 [28].

Kaplan-Meier plots were generated and differences in survival were evaluated using the log-rank test. Hazard ratios (HR) with 95 % confidence intervals (CI) were calculated using Cox proportional hazards regression models. High-proliferation groups were used as the reference category, with a hazard ratio below one indicating a benefit for patients with lower proliferation levels. PgR status at baseline, tumor size at baseline, and age (categorized into ≤ 40 and > 40 years) were included as covariates in multivariable Cox regression models. The age cut-off of 40 years is based on findings from the SOFT/TEXT trials, which indicate that younger patients tend to have more aggressive disease biology and poorer 12-year overall survival [29,30].

Spearman’s correlation test was used to evaluate the relationship between the continuous variables AURKA score (AS) and IHC-Ki67. Additionally, the Mann-Whitney U test was performed to determine whether the distribution of AS differed significantly between sub-groups defined by the dichotomized SSP-Ki67 and IHC-Ki67 scores. Kappa statistics was used to evaluate the agreement between IHC-Ki67 and SSP-Ki67 at specific time points while the McNemar's test was used to evaluate the significance of treatment-induced switches of dichotomized Ki67 status between different time points. Associations between baseline proliferation markers and pCR were assessed using Fisher’s exact test. All statistical tests were 2-sided and *p* < 0.05 was considered to be significant.

### Evaluation of tumor size response

A radiological assessment of the tumor was performed at baseline. Radiological tumor size was determined using ultrasound when available, otherwise by mammography. Post-surgery, histopathological measurements were used for the most accurate assessment of NACT effect on tumor size, since ultrasound was not performed after NACT completion before surgery. To assess the link between proliferation and tumor size dynamics, residual tumors were classified as responders versus non-responders using a cut-off of >30 % reduction of pre-treatment tumor size, to align with Response Evaluation Criteria in Solid Tumors (RECIST) criteria version 1.1 [31].

### Software

All data cleaning, integration, and analyses were conducted using RStudio version 2024.09.1 [32].

## Results

Between 2010 and 2019, *n* = 610 women with breast tumors of all clinical subtypes in the SCAN-B cohort (release #5) were scheduled to receive NACT. Of these, *n* = 175 patients presented with ER+/HER2- tumors and were eligible for inclusion in our analysis (Fig. 1). Baseline patient and tumor characteristics are summarized in Table 1. Patients were treated with anthracycline- and taxane-based regimens preoperatively and received postoperative endocrine therapy per Swedish guidelines at the time. At diagnosis, most tumors showed high IHC-Ki67 (96 %) and PgR positivity (82 %), with nodal involvement in 88 % of cases. The majority of patients (147/175; 84 %) were older than 40 years.

**Table 1:** Baseline characteristics of the patients included in this study.

	Summary
	*N* = 175
**Age, >40 or ≤40 years, n (%)**	
> 40	147 (84 %)
≤ 40	28 (16 %)
**Tumor Size, Median (range)**	30 (9-120)
**Pre-NACT Nodal Status, n (%)**	
N-	19 (13 %)
*N*+	133 (88 %)
**Pre-NACT SSP-Ki67 Status, n (%)**	
High	116 (70 %)
Low	50 (30 %)
Pre-NACT IHC-Ki67 Status*, n (%)	
High	164 (96 %)
Low	7 (4.1 %)
**Pre-NACT PgR Status, n (%)**	
Negative	32 (18 %)
Positive	142 (82 %)

Ki67 % ≥ 10 was considered high according to a predefined study-specific cut-off.

SSP: Single Sample Predictor, NACT: neoadjuvant chemotherapy.

Among the *n* = 175 patients with ER+/HER2- tumors, 86 (49 %) had a single sample successfully analyzed with good-quality RNAseq data: 81 from baseline (BL), 1 from cycle 2 (C2), and 4 from surgical specimens (Surg). Additionally, 76 patients (44 %) had two paired samples: 29 from BL and C2, 47 from BL and Surg, and none with a C2 and Surg combination. Only 9 patients (5 %) had paired samples from all three time points (BL, C2, and Surg). Lastly, 4 patients (2 %) had no samples with good-quality RNAseq data. One hundred and seventy-one patients (171/175, 98 %) had valid baseline IHC-Ki67 data, 152 (87 %) had valid IHC-Ki67 values from surgical specimens, and 149 (85 %) had valid paired IHC-Ki67 data for baseline and surgical specimens.

Only 10 (5.7 %) patients with ER+/HER2- tumors achieved a pCR following NACT, while 24 (14 %) experienced a relapse or died from breast cancer within a median follow-up time of 5.4 years.

### Correlation between GEA and IHC in assessing tumor proliferation status at different timepoints

**Fig. 2:**
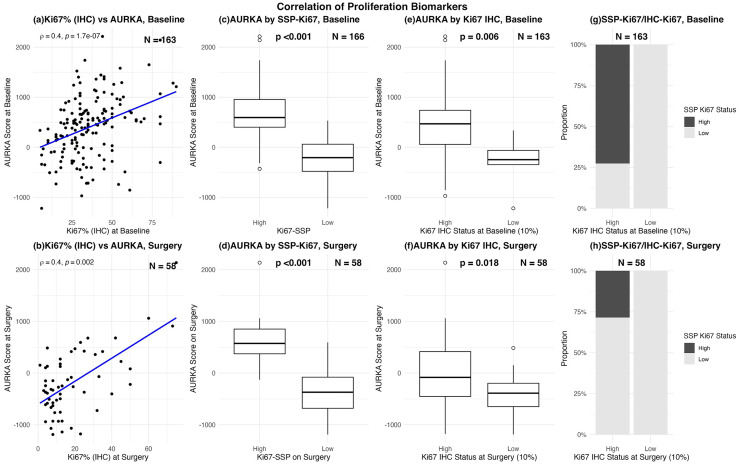
**Correlation between proliferation biomarkers**. (a–b) Scatter plots showing the relationship between IHC-Ki67 % (x-axis) and AURKA score (y-axis) at baseline (a) and surgery (b). Each dot represents a tumor sample; blue line represents linear fit. Annotated values show Spearman’s rank correlation coefficient (ρ) and associated two-sided test p-values, with *p* < 0.05 for both plots, indicating a statistically significant monotonic relationship. (c–f) Boxplots comparing AURKA scores (y-axis) between tumors classified as high versus low by SSP-Ki67 (c & d) or IHC-Ki67 status (e & f) at baseline (c, e) and surgery (d, f). Mann–Whitney U test was used to assess statistical significance, with p-values shown above plots. (g–h) Stacked bar plots illustrating the distribution of SSP-Ki67 classifications (dark grey = high, light grey = low) within tumors stratified by IHC-Ki67 status (10 % cut-off) at baseline (g) and surgery (h). Bars represent proportions of cases in each category.

A moderate correlation (Spearman’s ρ = 0.4) was observed between continuous IHC-Ki67 percentage scores and the paired AS proliferation score at both baseline (Fig. 2a) and in the residual tumors (Fig. 2b). Similarly, AS scores were consistently higher among tumors classified as “high” vs. “low” by both SSP-Ki67 (Fig. 2c, d) and IHC-Ki67 (Fig. 2e, F) at both baseline and surgery (Mann–Whitney *p* < 0.05). However, the difference in median AS scores was greater with SSP-Ki67 (Fig. 2c, d) compared to IHC-Ki67 (Fig. 2e, F).

At baseline, IHC-Ki67 classified more tumors as highly proliferative 96 % (157/163) compared to 70 % (116/166) with SSP-Ki67, with a pairwise agreement of 73.6 % (κ = 0.16) between the methods. Notably, all discordant cases (43/43) were classified as IHC—High versus SSP-Low, as shown in Fig. 2g and detailed in Supplementary Table S1. Next, the >20 % cutoff for IHC-Ki67 was explored, which corresponds to the IHC threshold implemented for training SSP-Ki67 signature. The percentage of cases classified as highly proliferative decreased from 96 % to 81 % (132/163) thereby improving the agreement with SSP-Ki67 to 75.5 % (κ = 0.35, Supplementary Table S1). Even with the 20 % cutoff, the majority of discordant cases (73 %, 29 out of 40) were classified as IHC—High and SSP-Low.

In surgical specimens, IHC-Ki67 again classified a larger proportion of tumors as highly proliferative relative to SSP-K67. Using the 10 % cutoff, 60 % (35/58) were classified as highly proliferative by IHC-Ki67 compared to only 17 % (10/58) by SSP-Ki67, resulting in a pairwise agreement of 56.9 % (κ = 0.24) between the methods. All IHC-low cases were consistently classified as lowly proliferative by SSP-Ki67, while 71 % (25/35) of the IHC—High cases were discordantly classified as SSP-Low, as illustrated in Fig. 2h and summarized in Supplementary Table S2. When applying the >20 % IHC cut-off, only 29 % (17/58) of tumors were highly proliferative by IHC, with 53 % (9/17) discordantly classified as lowly proliferative by SSP-Ki67, increasing the overall agreement between the methods to 81.0 % (κ = 0.48, Supplementary Table S2).

### Effect of NACT on tumor proliferation

**Fig. 3:**
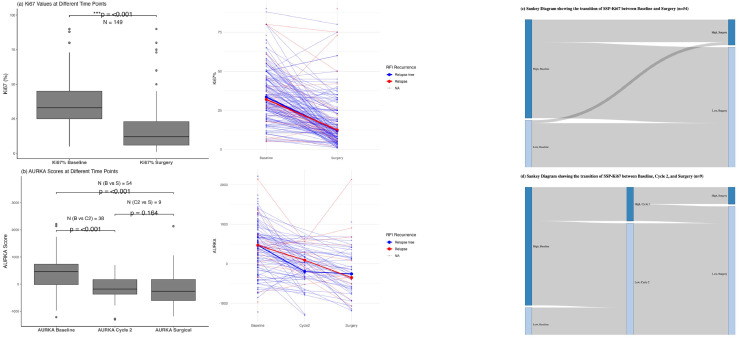
**Dynamics of Proliferation biomarkers during NACT**. (a) IHC-Ki67 % decreased significantly from baseline (median = 33 %) to surgery (median = 12 %; *p* < 0.001, Wilcoxon signed-rank test). The boxplot (left) shows distributions, and the spaghetti plot (right) illustrates individual patient trajectories, color-coded by recurrence-free interval (RFI). Bold lines indicate subgroup medians. (b) AURKA scores decreased significantly from baseline to cycle 2 and from baseline to surgery (*p* < 0.001), with no significant difference between cycle 2 and surgery (*p* = 0.164). The boxplot (left) shows group medians, and the spaghetti plot (right) shows individual trajectories by RFI outcome, with bold lines indicating subgroup medians. (c) Sankey diagram showing transitions in SSP-Ki67 classification (high vs. low) from baseline to surgery . Of 35 tumors classified as high at baseline, 27 transitioned to low at surgery. (d) Sankey diagram depicting SSP-Ki67 trajectories across baseline, cycle 2, and surgery in the subset of patients with paired mRNA data. All tumors that transitioned from high to low at cycle 2 remained low at surgery.

Among patients (*n* = 149) with paired IHC-Ki67 data at baseline and surgery, the median IHC-Ki67 percentage score at baseline was 33 %, which significantly decreased after NACT to 12 % among residual tumors (Fig. 3a, Wilcoxon paired test, *p* < 0.001). Proliferation status by IHC-Ki67 changed in 36 % (54/149) of tumors following NACT; with 98 % (53/54) shifting from high proliferation at baseline to low proliferation at surgery (McNemar’s *p* < 0.001, Supplementary Table S3).

Comparable trends were observed in the analysis of AS dynamics. NACT induced a significant reduction in AS between baseline and cycle 2 (C2) (Wilcoxon paired test, *p* *<* *0*.001, Fig. 3b) and between baseline and surgical specimens (Wilcoxon paired test, *p* *<* *0*.001; Fig. 3b), respectively. In contrast, median AS between C2 and surgery were similar (Wilcoxon paired test, p = 0.164, Fig. 3b), suggesting that proliferation levels stabilized following the initial response seen after two cycles of NACT.

Paired SSP-Ki67 data were available for 54 patients for baseline versus surgery comparisons, and for 38 patients for baseline versus C2 comparisons. At baseline, 65 % (35/54) were classified highly proliferative. Following 6 cycles of NACT, 77 % (27/35) of highly proliferative baseline tumors were reclassified as low proliferative in the surgical specimen (McNemar's *p* < 0.001; Fig. 3 and Supplementary Table S4). Similarly, after NACT cycle 2 (C2), 42 % (16/38) showed a shift in proliferation status; all drifting from high proliferation at baseline to low proliferation after cycle 2 (McNemar's *p* < 0.001; Supplementary Table S5).

In a smaller subset of nine tumors with paired SSP-Ki67 data across all three time points (baseline, C2, and surgery), dynamic changes in proliferation status were observed, consistent with previous findings, as shown in Fig. 3d Five tumors transitioned from high proliferation at baseline to low at C2 and remained low at surgery, consistent with AS proliferation dynamics. In three cases, baseline proliferation status was not affected by treatment: two remained consistently lowly proliferative across all time points, and one remained consistently highly proliferative. One additional case maintained high proliferation between baseline and C2 but shifted to low at surgery. Between C2 and surgery, 7/9 tumors were consistently classified as lowly proliferative, one remained highly proliferative, and one switched from high to low proliferation. However, consistent with AS proliferation dynamics, the change in proliferation status between C2 and surgery was not statistically significant (McNemar’s *p* = 1; Supplementary Table S6).

### Proliferation at baseline in relation to prognosis and pCR

**Fig. 4:**
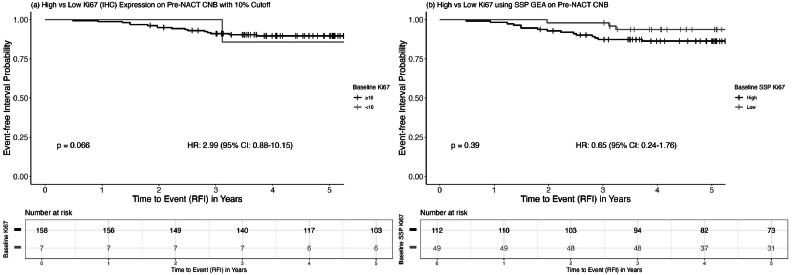
Kaplan–Meier curves showing the association of baseline proliferation with RFI: (a) IHC-Ki67 using a 10 % cut-off and (b) SSP-Ki67. P-values are based on the log-rank test.

Baseline proliferation, assessed by IHC-Ki67 was initially examined for its prognostic value. The analysis suggested an increased risk of relapse for tumors classified as low proliferative (HR = 2.99, 95 % CI: 0.88–10.15; Fig. 4a). However, this finding should be interpreted with caution, as the low-proliferative subgroup was small (*n* = 7) and only one relapse was recorded. The uneven distribution reflects the patient selection for NACT, which inherently favored the inclusion of higher-proliferative tumors. We therefore do not regard this observation as providing reliable information of prognostic value. The prognostic value of baseline proliferation assessed by SSP-Ki67 was next evaluated. While lower proliferation seemed to be linked to a better prognosis, the results were not statistically significant (SSP-Ki67: HR = 0.65, 95 %CI: 0.24–1.76), as shown in Fig. 4b

After adjusting for baseline PgR status, tumor size, and age at diagnosis, the association between baseline proliferation and RFI remained non-significant IHC-Ki67 (HR = 1.76, 95 %CI: 0.23–13.50) and SSP-Ki67(HR = 0.65, 95 %CI: 0.21–2.03).

High baseline proliferation was associated with pCR. All pCR events occurred in patients classified as highly proliferative on baseline, using both SSP-Ki67 and IHC-Ki67. Specifically, pCR was achieved in 10/116 (8.6 %) patients classified as high by SSP-Ki67, 10/140 (7.1 %) by IHC-Ki67 using a 20 % cut-off, and 10/164 (6.1 %) by IHC-Ki67 using a 10 % cut-off. Only SSP-Ki67 was significantly associated with pCR (*p* = 0.033, Fisher’s exact test), whereas IHC-Ki67 using 10 % and 20 % cut offs was not (*p* = 1.0 and *p* = 0.211, respectively, Fisher’s exact test). This difference likely reflects the fact that SSP-Ki67 classified fewer patients as highly proliferative compared with IHC-Ki67.

### Proliferation after two cycles of NACT in relation to prognosis

**Fig. 5:**
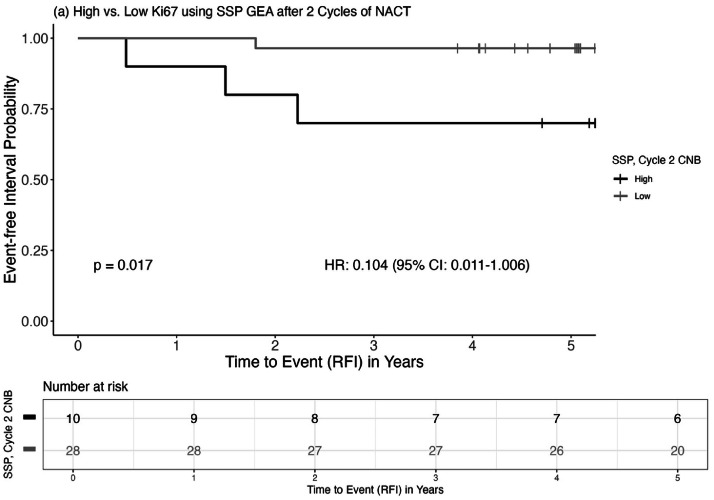
Kaplan–Meier curve of RFI stratified by high versus low SSP-Ki67 in biopsies after two cycles of NACT. P-value is based on the log-rank test.

The prognostic value of interim proliferation status, assessed after two cycles of NACT by SSP-Ki67, was next evaluated. Patients with low-proliferative C2 tumors, as determined by SSP-Ki67, showed a borderline significant trend toward improved prognosis in univariable analysis (SSP-Ki67: HR = 0.104, 95 % CI: 0.011–1.006, Log-rank *p* = 0.017; Fig. 5). This trend persisted after adjusting for baseline PgR status, tumor size, and age (SSP-Ki67: HR = 0.38, 95 % CI: 0.03–4.15), although non-significant, likely due to the small number of cases included in the analysis.

One of the 10 patients who achieved a pCR had paired biopsies at baseline and cycle 2. A shift from high SSP-Ki67 at baseline to low SSP-Ki67 after cycle 2 was observed for this patient.

### Proliferation after six cycles of NACT in relation to prognosis

**Fig. 6:**
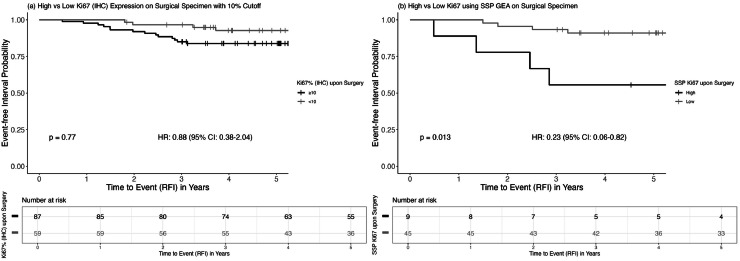
Kaplan–Meier curves illustrating (a) RFI according to Ki67 expression (≥10 % vs. <10 %) in surgical specimens and (b) RFI according to SSP-Ki67 proliferation levels in surgical specimens. P-values are based on the log-rank test.

High proliferation assessed upon surgery following six cycles of NACT was identified as a negative prognostic biomarker. Using IHC, low Ki67 (<10 %) was associated with a reduced risk of recurrence, with a HR of 0.88 (95 %CI: 0.38–2.04, Fig. 6a) and a corresponding HR of 0.71 (95 %CI: 0.26–1.94) after adjusting for baseline PgR status, tumor size, and age.

Similarly, when SSP-Ki67 was utilized, high proliferation following complete NACT remained a negative prognostic biomarker. Low proliferation was associated with a decreased risk for RFI events in the univariable analysis (HR = 0.23, 95 %CI: 0.06–0.82, Fig. 6b) and HR = 0.05, (95 %CI: 0.01–0.42) after adjusting for baseline PgR status, tumor size, and age.

### Dynamic NACT-induced changes in proliferation in relation to prognosis

Considering our previous observation that the impact of brief exposure to NACT on proliferation persisted after completing NACT treatment, we included in this analysis any sample collected after the initiation of NACT i.e. either after C2 or surgery. In cases where data from both C2 and surgical specimens were available for the same patient, the C2 information was prioritized to capture the impact of early NACT-induced changes. Dynamic change in proliferation was captured by sub-categorizing patients by combining baseline and treatment-exposed status of the binary (low vs. high) Ki67 categories as follows: low-low (LL), low-high (LH), high-low (HL), and high-high (HH).

**Fig. 7:**
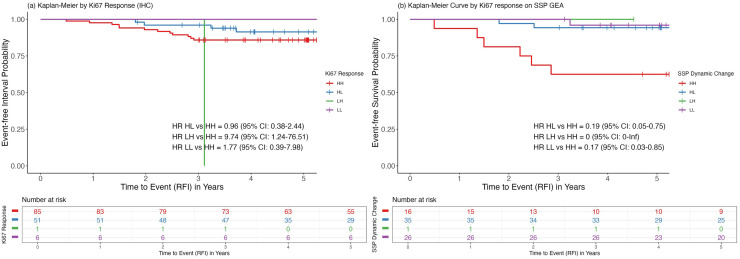
Kaplan–Meier curves illustrating the association of dynamic changes in proliferation with RFI using (a) IHC-Ki67 with a 10 % cut-off and (b) SSP-Ki67. Proliferation dynamics are defined as follows: HH, high to high; HL, high to low; LH, low to high; LL, low to low.

Using IHC-Ki67, a treatment-induced reduction in proliferation (HL) was associated with a more favorable prognosis compared to a persistent high proliferation (HH) after exposure to NACT (Fig. 7a; HR=0.96,95 %CI: 0.38–2.44, *p* = 0.93), and the difference remained statistically insignificant after adjusting for baseline PgR status, tumor size, and age (HR=0.85, 95 %CI: 0.31–2.34, *p* = 0.75).

NACT-induced changes in proliferation assessed by SSP-Ki67 produced analogous findings. Conversion from high to low proliferation (HL) was prognostic of a favorable outcome, with a significantly lower risk for relapse compared to patients with persistently high proliferation (HH) (Fig. 6b; HR = 0.19, (95 %CI: 0.05–0.75, *p* = 0.018). However, this result lost statistical significance after adjusting for PgR status, tumor size and age at baseline (HR = 0.32, 95 %CI: 0.07–1.53, *p* = 0.16).

Dynamic changes in SSP-Ki67 between baseline and each time point (C2 and surgery) in relation to prognosis were further assessed as a sensitivity analysis. This analysis included all cases with paired SSP-Ki67 assessments at either cycle 2 or surgery. The association between a favorable prognosis and reduction in tumor proliferation rate appeared to be consistent both after cycle 2 (Supplementary Figure S1a; HR = 0.20, 95 %CI: 0.02–1.89) as well as at surgery (Supplementary Figure S1b, HR = 0.19, 95 %CI: 0.05–0.78). After adjusting for the baseline covariates PgR status, tumor size, and age, the results for C2 and surgery were HR = 1.45, (95 %CI: 0.13–16.10) and HR = 0.06, (95 %CI: 0.01–0.50), respectively.

### Tumor shrinkage and proliferation dynamics

Among *n* = 165 patients with residual disease, an anatomical response was defined as a 30 % reduction in tumor size, comparing baseline radiological size to post-treatment histology, to mimic the RECIST criteria. Optimally, the same modality should be used to evaluate tumor shrinkage. However, due to the absence of radiological data following NACT, histological assessment was used instead. This analysis remains exploratory.

There was no difference in anatomical response between tumors with persistently high proliferation and those with reduced proliferation. Patients achieving ≥30 % tumor size reduction could still have persistently high proliferative tumors and vice versa. Depending on the method used for assessing proliferation status, 60–69 % of patients showed >30 % tumor shrinkage despite having persistently high proliferation, as shown in Table 2. These findings indicate that a reduction in tumor proliferation does not always align with tumor shrinkage, implying that anatomic response does not necessarily reflect a favorable molecular biological response to NACT treatment in ER+/HER2- breast cancer.

**Table 2:** Association of tumor shrinkage with proliferation dynamics using IHC-Ki67 and SSP-Ki67. Radiological response was defined as ≥30 % reduction in tumor size, classified as a responder per RECIST criteria.

	Anatomical Response*	
	Non-responder *N* = 60^1^	Responder *N* = 68^1^
**Proliferation Response IHC-Ki67**		
**HH**	31 (40 %)	47 (60 %)
**HL**	26 (57 %)	20 (43 %)
**LH**	1 (100 %)	0 (0 %)
**LL**	2 (67 %)	1 (33 %)
**Proliferation Response SSP-Ki67**	**Non-responder** *N* = 29^1^	**Responder** *N* = 42^1^
**HH**	4 (31 %)	9 (69 %)
**HL**	14 (42 %)	19 (58 %)
**LH**	0 (NA %)	0 (NA %)
**LL**	11 (44 %)	14 (56 %)

⁎ According to RECIST criteria Proliferation dymanics: HH; high to high, HL; high to low, LH; low to high and LL; low to low.

1 n (%).

## Discussion

In this Swedish cohort of ER+/HER2- patients undergoing NACT, only 5.7 % achieved a pCR, which is similar to pCR rates reported in previous studies [33,34]. Despite the low pCR rate, only 24/175 patients (14 %) in the cohort experienced an RFI event within a median follow-up period of 5.4 years, underscoring the need for additional biomarkers that can predict favorable outcomes and guide treatment de-escalation decisions.

Our study could not show that baseline proliferation status was prognostic for RFI among patients planned for NACT, likely because of the very high selection (96 %) of high-proliferative tumors which underpowered the ability to detect differences in RFI between the high- and low-proliferation groups. In contrast, the POETIC trial demonstrated that patients with low baseline IHC-Ki67 rarely relapsed, and that 73 % of those with high baseline IHC-Ki67 converted to low IHC-Ki67 (HL) after two weeks of NET, experiencing an 8.4 % recurrence rate compared with 4.3 % among those with consistently low (LL) IHC-Ki67; patients with persistently high proliferation had a relapse rate of 21.5 % [16]. Together, these findings highlight that while baseline IHC-Ki67 may have prognostic value when NET is given, short-term changes in IHC-Ki67 provide greater prognostic insight.

Our study suggests that high baseline proliferation is associated with pCR. In line with this finding, previous studies have shown that high proliferation is predictive of pCR following NACT across all breast cancer subtypes[35], which is indicative of chemosensitivity. This chemosensitivity may offset the otherwise adverse prognostic impact of high baseline proliferation, helping to explain why baseline proliferation does not appear to be strongly prognostic. In contrast, assessment of proliferation after a brief period of therapy, whether NET in previous studies or NACT in our study, appears to identify tumors with treatment resistance, thereby enhancing the prognostic value of the biomarker.

Our findings suggest that dynamic changes in tumor proliferation, as assessed both by IHC and GEAs, can identify patients at higher risk for disease recurrence after preoperative chemotherapy. Persistent high proliferation following exposure to NACT emerged as a negative prognostic biomarker among patients with ER+/HER2- early breast cancers. A larger sample size may have yielded more statistically significant results. However, the uniformity of our findings across the different assays for tumor proliferation evaluation supports the hypothesis that treatment-induced reduction in tumor proliferation is a biomarker of a favorable prognosis. Notably, high proliferation assessed by SSP-Ki67 during or after treatment appeared to more accurately identify patients at high risk for relapse compared to IHC-KI67. External validation trials in sufficiently large cohorts are the next step for further proving this biomarker’s analytical and clinical validity. Similar findings have also been reported in studies across other breast cancer subtypes, where a reduction in IHC-Ki67 following neoadjuvant chemotherapy, anti-HER2 therapy, and endocrine treatment has been associated with a better prognosis [[36], [37], [38]]. Together, the results of these studies suggest that the prognostic utility of proliferation biomarkers may be enhanced when treatment is administered before assessing these biomarkers.

SSP-Ki67 consistently classified more tumors as low proliferative compared to IHC, using both 10 % and 20 % cutoffs for IHC-Ki67. SSP-Ki67 assessed on-treatment (after C2) and in the residual tumor performed better in identifying a small subgroup of patients with a significantly elevated risk for relapse. In contrast, IHC-Ki67 classified a larger proportion of residual tumors as highly proliferative, reducing its prognostic significance. These results suggest a risk for overtreatment when relying on IHC-Ki67, highlighting the strength of mRNA-based biomarkers, consistent with the role of prognostic gene signatures in the adjuvant setting, where they typically classify fewer patients as high genomic risk, thereby safely sparing them unnecessary toxic treatments [[3], [4], [5], [6], [7]].

When evaluating the impact of treatment on biomarker status, it is also essential to account for variability introduced by different sampling or analytical methods and tumor heterogeneity, which are all factors that can confound results. In a previous study by our group, we observed concordance rates of 82.9 % for IHC-Ki67 (using a 20 % cutoff) and 86.4 % for SSP-Ki67 respectively between paired baseline CNB and treatment-naïve surgical specimens, and most discrepancies were associated with a shift from low proliferation in the CNB to high proliferation in the surgical resection [18]. In contrast, the current analyses demonstrate significant conversion from high proliferation at baseline to low proliferation in the residual tumor, indicating a treatment effect consistent with the expected anti-proliferative impact of chemotherapeutic agents and aligns with the favorable association with prognosis. Still, the impact of technical variability and tumor heterogeneity cannot be excluded from our findings.

As noted in previous studies, the 10 % cut-off used to separate low versus high proliferation seems arbitrary, hence other Ki67 cut-offs warrant further exploration. Nonetheless, this 10 % cut-off is the most well-established cut-off used in related studies investigating the significance of treatment-induced changes in tumor proliferation, including the POETIC, ADAPT, and ALTERNATE trials [14,15,39]. The International Ki67 Working Group (IKWG) suggests using cut-offs of ≤5 % for low proliferation and ≥30 % for high proliferation for estimating prognosis in ER+/HER2- T1-T2, N0-N1 breast cancer[17]. However, in our cohort, more than half of the tumors have intermediate Ki67 % values (between 6–29 %) both at baseline and at surgery, reflecting a clear limitation of these cut-offs.

Our results align with previous studies on the effect of NACT on proliferation in early breast cancer. In a study by Sinn et al., persistent or increased levels of IHC-Ki67 in an on-treatment biopsy during NACT was associated with a lower pCR rate and poorer disease-free survival in TNBC [40]. However, this association was not found to be significant in HR+/HER2− breast cancer [40]. Similarly, Turnbull et al. [41] reported that proliferation-associated genes were more significantly down-regulated in on-treatment biopsies of NET responders compared to non-responders. Furthermore, it was shown that assessing proliferation after two weeks of treatment enhances the accuracy of response prediction, supporting the role of early treatment-induced changes in proliferation as a predictive marker of therapeutic response [41].

Despite some limitations, our findings highlight the potential value of the SSP-Ki67 biomarker in refining risk stratification among patients with ER+/HER2- breast cancer undergoing NACT. Although this breast cancer subtype generally carries a favorable prognosis, there remains a strong clinical need for more individualized treatment approaches. Our results suggest that SSP-Ki67 can help identify patients at higher risk of recurrence despite receiving NACT, offering an opportunity to tailor adjuvant therapy more precisely to individual risk profiles. For example, if mRNA-based proliferation were used to identify high-risk patients for adjuvant CDK4/6 inhibitors instead of conventional IHC-Ki67, SSP-Ki67 would have classified only 9 of 54 patients (16.7 %) as highly proliferative and thus high-risk. By contrast, IHC-Ki67 classified 87 of 146 patients (59.6 %) as high-risk. While only a randomized controlled trial can determine whether it is safe to omit CDK4/6 inhibitors in patients who switch to low proliferation following neoadjuvant treatment, the high number needed to treat, together with reliance on anatomic risk staging (e.g., nodal status), suggests it is reasonable to explore more specific biomarkers of favorable prognosis. Such biomarkers could better identify truly high-risk patients, reduce overtreatment, and potentially enable less invasive approaches, such as avoiding axillary nodal dissection [42,43].

One important limitation of our study is the relatively small cohort size (*n* = 175). The small number of patients, in combination with a low number of outcome events and missing biomarker data at certain time points, may have reduced the statistical power to detect some biologically and clinically meaningful associations. Future studies with larger sample sizes are needed to more robustly assess these associations. Another limitation is the relatively short median follow-up time (5.4 years), which is particularly relevant for ER+/HER2- breast cancer given its well-known tendency for late relapse. However, despite these limitations, and considering the generally favorable prognosis of ER+/HER2- breast cancer, the need for tailoring treatment cannot be ignored. Our findings suggest that the SSP-Ki67 biomarker has good potential for identifying patients at higher risk of recurrence, despite receiving NACT, offering the possibility to tailor adjuvant treatment according to the patient’s individual risk.

Dynamic assessment of proliferation appears to be a promising method to enable response-adapted therapy. For example, future clinical trials could investigate the safety of omitting additional NACT cycles in patients whose tumors show a significant decrease in proliferation after a brief exposure (e.g. 2 cycles) to treatment. Similarly, future trials could evaluate whether patients with reduced proliferation after preoperative treatment might safely forgo lengthy postoperative CDK4/6 inhibitor treatments. A similar principle proved to be successful in the German ADAPT trial, which used the IHC-Ki67 response after three weeks of preoperative endocrine therapy to guide decisions on treatment de-escalation by omission of adjuvant chemotherapy. In the ADAPT trial, patients who demonstrated a favorable IHC-Ki67 response to NET and who would have otherwise been planned for chemotherapy by clinical risk stratification were able to safely forgo chemotherapy, achieving outcomes comparable to those who received chemotherapy without exposure to toxic treatment [14]. These findings, along with emerging clinical evidence, could have a practice-changing impact by enabling more precise response-adapted treatment using dynamic proliferation assessment, ultimately leading to better-designed clinical trials.

## Conclusion

Tumor proliferation status can be used as a prognostic biomarker for the risk of disease relapse when measured after brief exposure to NACT or in the residual tumor after NACT. GEA-based proliferation markers outperform IHC in identifying patients with a poorer prognosis while consistently classifying fewer patients as highly proliferative. These biomarkers may enable safe de-escalation of treatment, reducing overtreatment. Dynamic assessment of proliferation can aid in tailoring treatment strategies, enabling response-adapted treatment, which can be implemented in clinical trials of preoperative chemotherapy in early luminal breast cancer.

## Funding

Financial support for this study was through research grants provided by the Mrs Berta Kamprad Foundation (FBKS 2022-35 and FBKS-2020-9), the Gunnar Nilsson’s Foundation (GN-2022-7-283), the Swedish Research Council (2018–00522) and Swedish governmental funding (ALF, grant to N.L.). Funding organizations did not participate in the actual research activities.

## CRediT authorship contribution statement

**Hani Saghir:** Writing – review & editing, Writing – original draft, Visualization, Validation, Software, Resources, Project administration, Methodology, Investigation, Formal analysis, Data curation, Conceptualization. **Srinivas Veerla:** Writing – review & editing, Supervision, Resources, Formal analysis. **Niklas Loman:** Writing – review & editing, Supervision, Resources, Project administration, Methodology, Investigation, Funding acquisition, Formal analysis, Conceptualization. **Siker Kimbung:** Writing – review & editing, Visualization, Validation, Supervision, Resources, Project administration, Methodology, Investigation, Funding acquisition, Formal analysis, Data curation, Conceptualization.

## Declaration of competing interest

The authors declare that they have no known competing financial interests or personal relationships that could have appeared to influence the work reported in this paper.

## Appendix Supplementary materials

Image, application 1
