# Supplementary material for: Dynamic assessment of proliferation to guide response-adapted therapy in the setting of neoadjuvant chemotherapy in ER+/HER2- breast cancer
Source: Transl Oncol. 2025 Nov 15;63:102597. doi: 10.1016/j.tranon.2025.102597 (PMC12663844; doi:10.1016/j.tranon.2025.102597)
Supplement: Supplementary file 1 [file mmc1.pptx]

## Slide 1
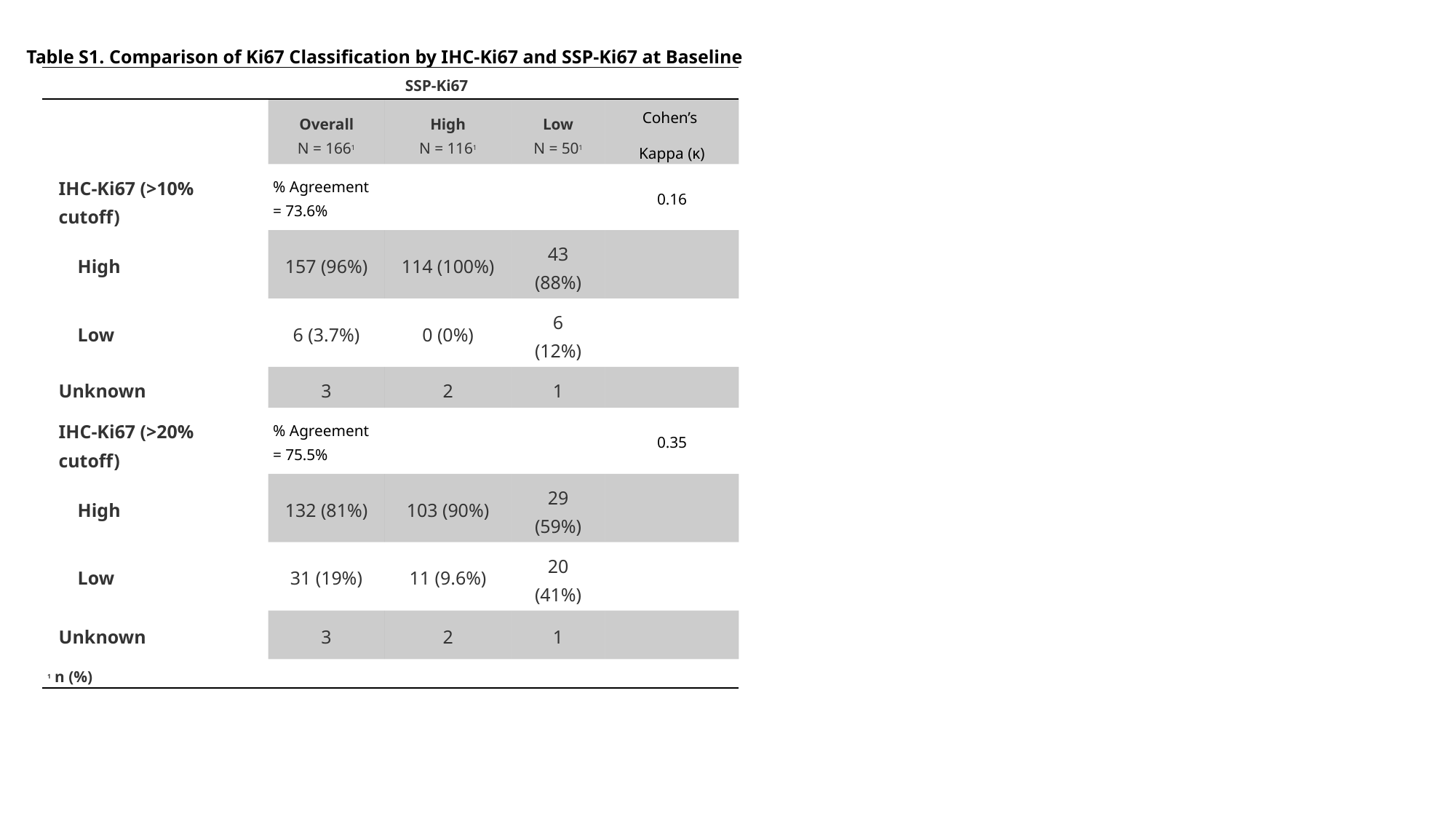

Table S1. Comparison of Ki67 Classification by IHC-Ki67 and SSP-Ki67 at Baseline
| | SSP-Ki67 | | | |
| --- | --- | --- | --- | --- |
| | Overall N = 1661 | High N = 1161 | Low N = 501 | Cohen’s Kappa (κ) |
| IHC-Ki67 (>10% cutoff) | % Agreement = 73.6% | | | 0.16 |
| High | 157 (96%) | 114 (100%) | 43 (88%) | |
| Low | 6 (3.7%) | 0 (0%) | 6 (12%) | |
| Unknown | 3 | 2 | 1 | |
| IHC-Ki67 (>20% cutoff) | % Agreement = 75.5% | | | 0.35 |
| High | 132 (81%) | 103 (90%) | 29 (59%) | |
| Low | 31 (19%) | 11 (9.6%) | 20 (41%) | |
| Unknown | 3 | 2 | 1 | |
| 1 n (%) | | | | |

## Slide 2
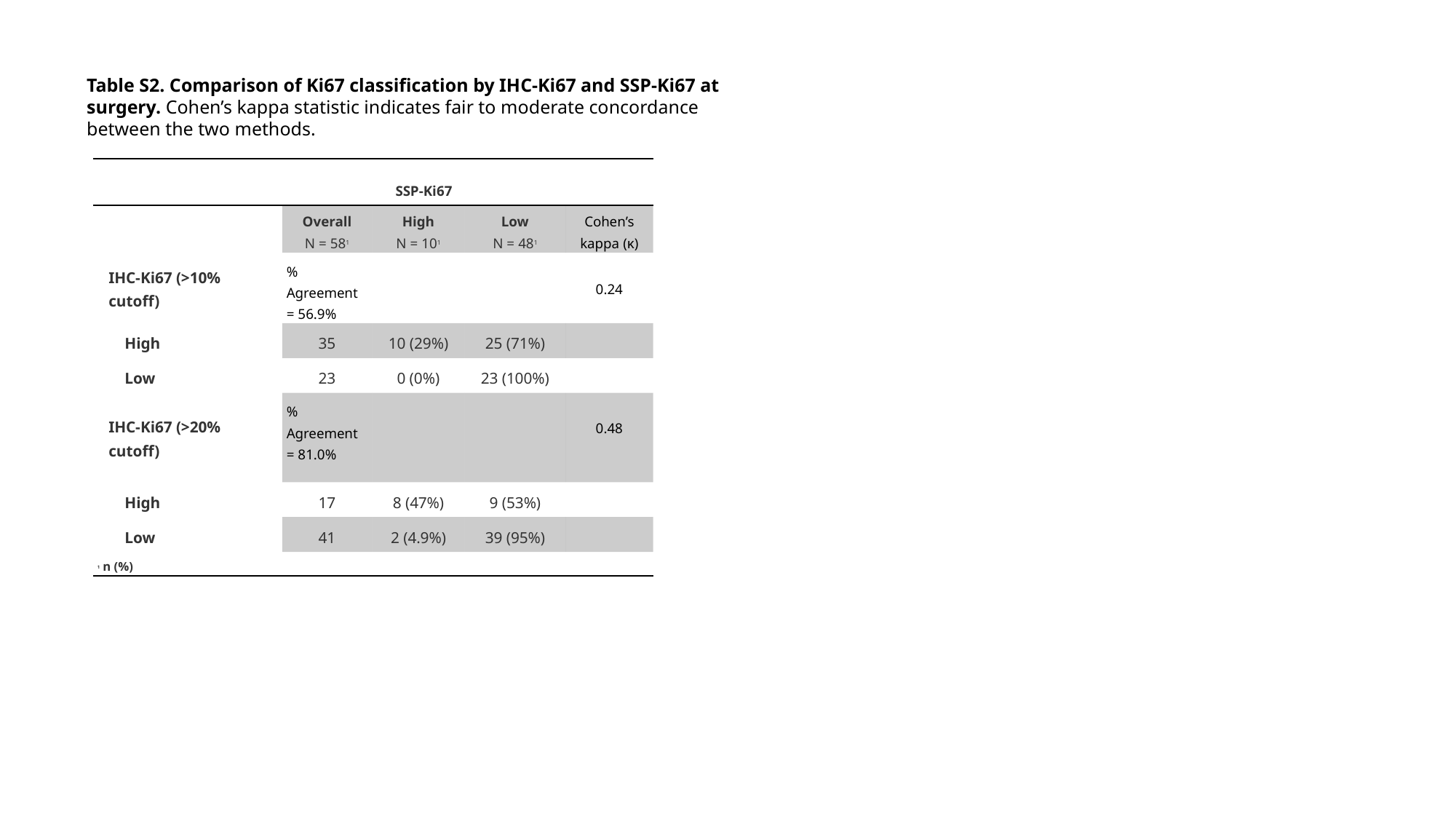

Table S2. Comparison of Ki67 classification by IHC-Ki67 and SSP-Ki67 at surgery. Cohen’s kappa statistic indicates fair to moderate concordance between the two methods.
| | SSP-Ki67 | | | |
| --- | --- | --- | --- | --- |
| | Overall N = 581 | High N = 101 | Low N = 481 | Cohen’s kappa (κ) |
| IHC-Ki67 (>10% cutoff) | % Agreement = 56.9% | | | 0.24 |
| High | 35 | 10 (29%) | 25 (71%) | |
| Low | 23 | 0 (0%) | 23 (100%) | |
| IHC-Ki67 (>20% cutoff) | % Agreement = 81.0% | | | 0.48 |
| High | 17 | 8 (47%) | 9 (53%) | |
| Low | 41 | 2 (4.9%) | 39 (95%) | |
| 1 n (%) | | | | |

## Slide 3
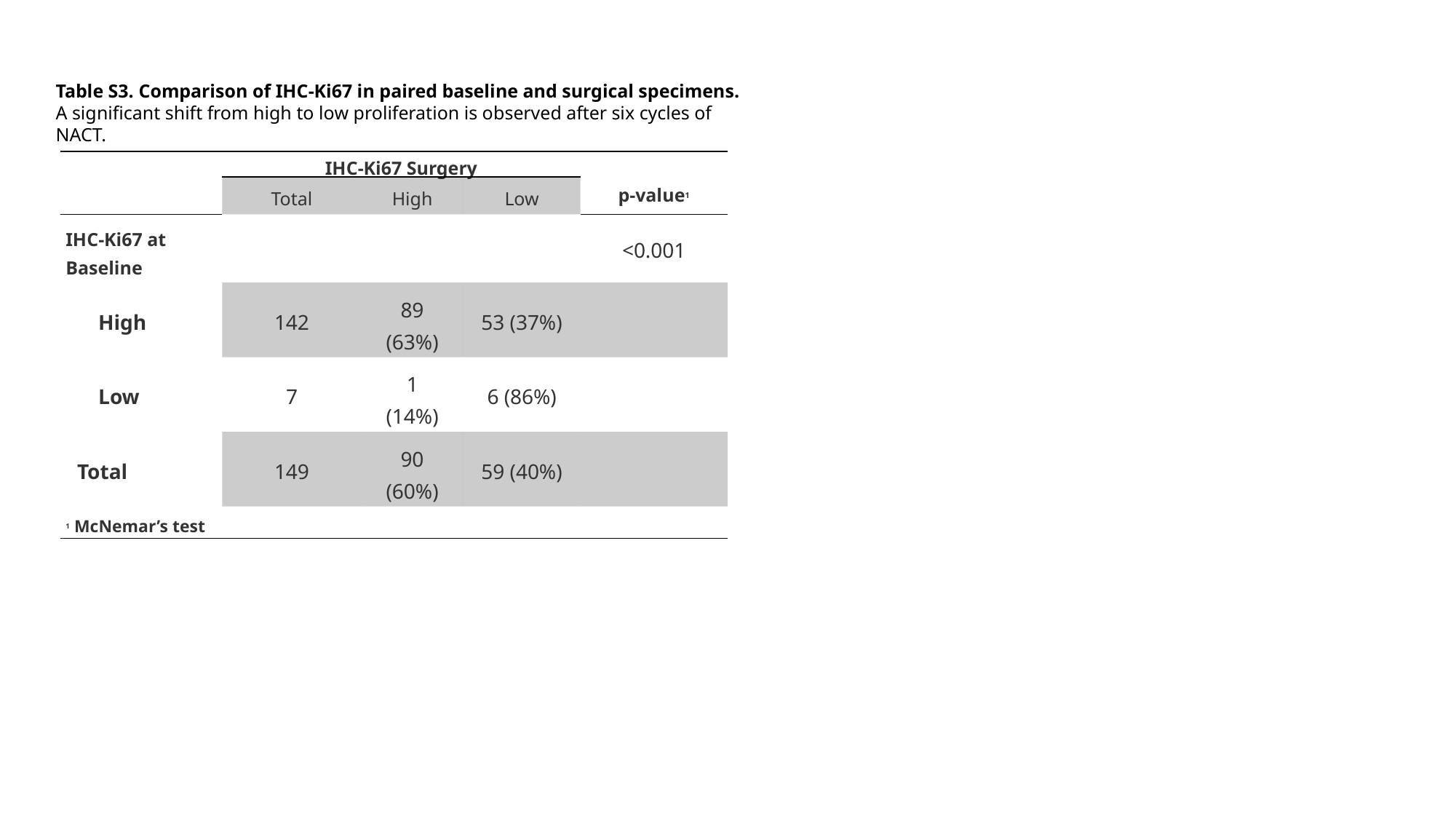

Table S3. Comparison of IHC-Ki67 in paired baseline and surgical specimens. A significant shift from high to low proliferation is observed after six cycles of NACT.
| | IHC-Ki67 Surgery | | | p-value1 |
| --- | --- | --- | --- | --- |
| | Total | High | Low | |
| IHC-Ki67 at Baseline | | | | <0.001 |
| High | 142 | 89 (63%) | 53 (37%) | |
| Low | 7 | 1 (14%) | 6 (86%) | |
| Total | 149 | 90 (60%) | 59 (40%) | |
| 1 McNemar’s test | | | | |

## Slide 4
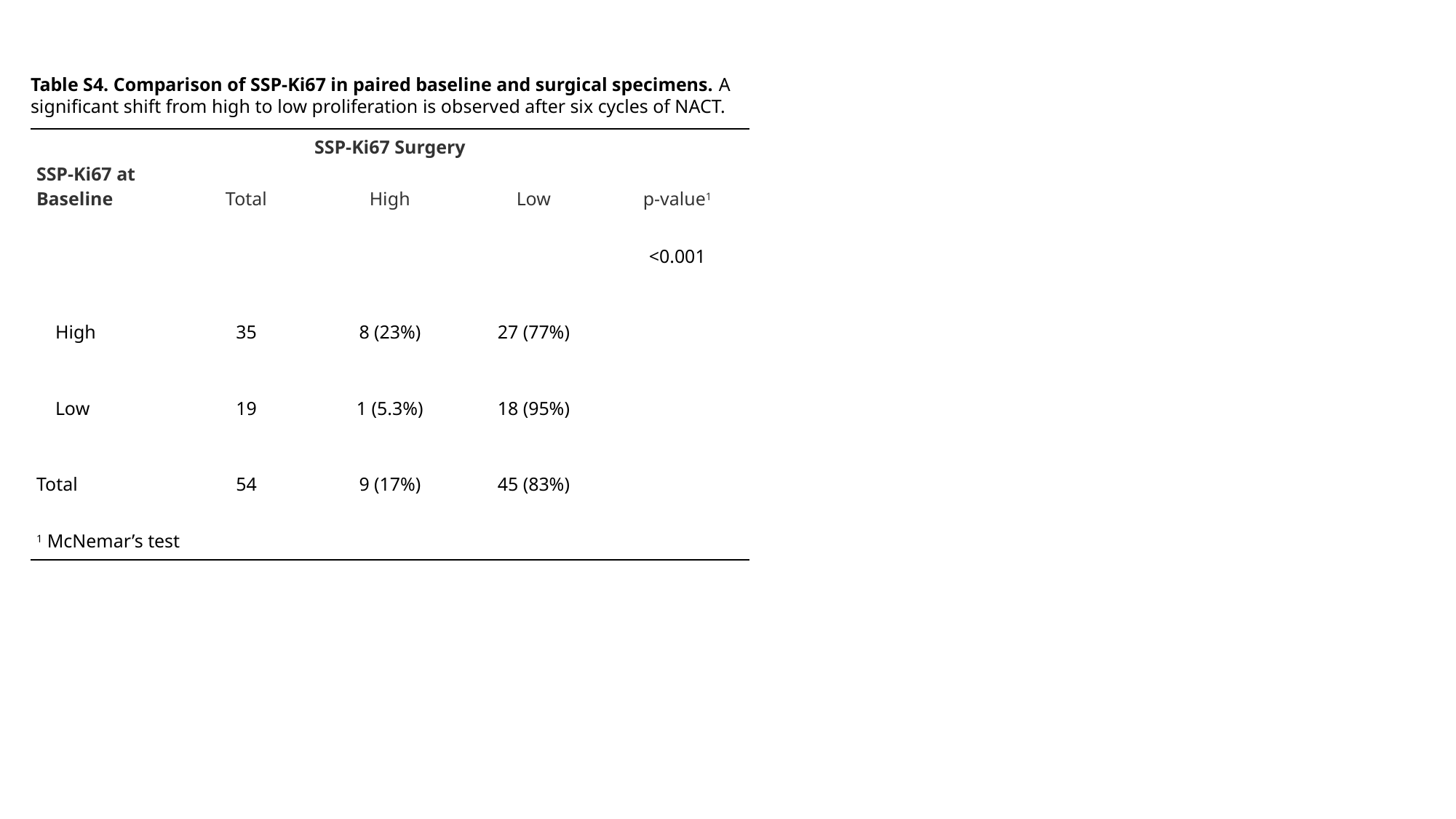

Table S4. Comparison of SSP-Ki67 in paired baseline and surgical specimens. A significant shift from high to low proliferation is observed after six cycles of NACT.
| SSP-Ki67 at Baseline | SSP-Ki67 Surgery | | | p-value1 |
| --- | --- | --- | --- | --- |
| | Total | High | Low | |
| | | | | <0.001 |
| High | 35 | 8 (23%) | 27 (77%) | |
| Low | 19 | 1 (5.3%) | 18 (95%) | |
| Total | 54 | 9 (17%) | 45 (83%) | |
| 1 McNemar’s test | | | | |

## Slide 5
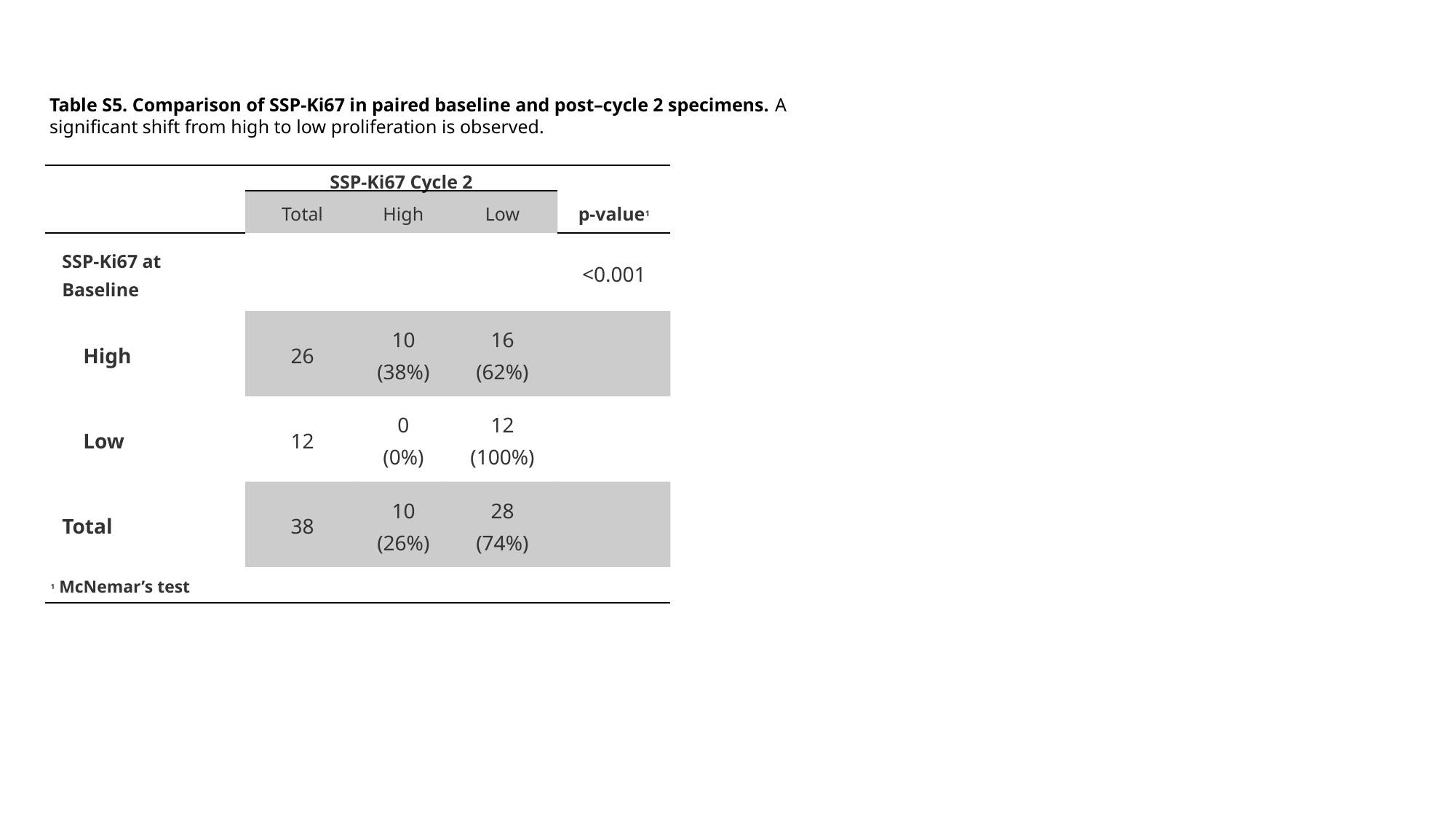

Table S5. Comparison of SSP-Ki67 in paired baseline and post–cycle 2 specimens. A significant shift from high to low proliferation is observed.
| | SSP-Ki67 Cycle 2 | | | p-value1 |
| --- | --- | --- | --- | --- |
| | Total | High | Low | |
| SSP-Ki67 at Baseline | | | | <0.001 |
| High | 26 | 10 (38%) | 16 (62%) | |
| Low | 12 | 0 (0%) | 12 (100%) | |
| Total | 38 | 10 (26%) | 28 (74%) | |
| 1 McNemar’s test | | | | |

## Slide 6
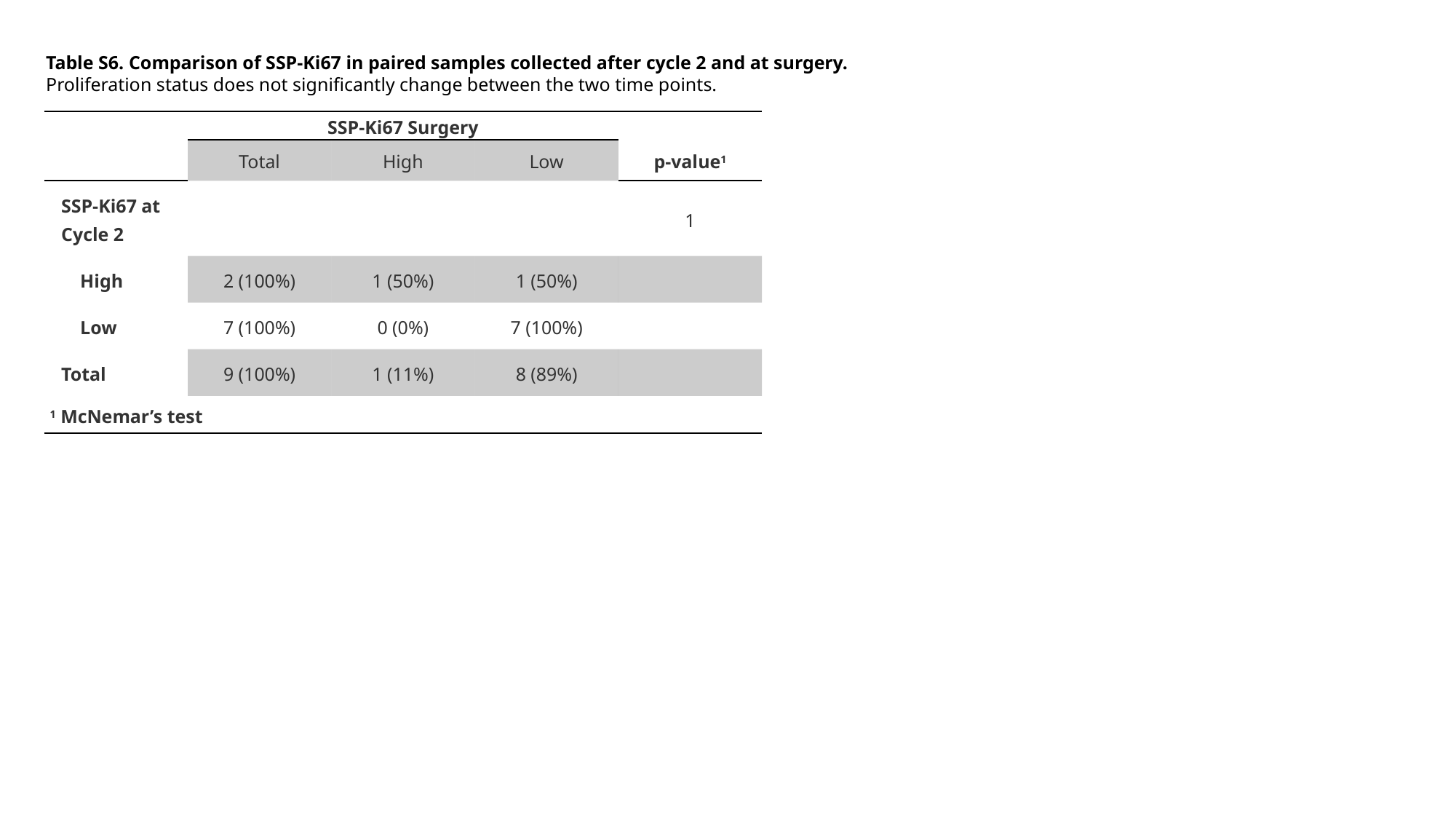

Table S6. Comparison of SSP-Ki67 in paired samples collected after cycle 2 and at surgery. Proliferation status does not significantly change between the two time points.
| | SSP-Ki67 Surgery | | | p-value1 |
| --- | --- | --- | --- | --- |
| | Total | High | Low | |
| SSP-Ki67 at Cycle 2 | | | | 1 |
| High | 2 (100%) | 1 (50%) | 1 (50%) | |
| Low | 7 (100%) | 0 (0%) | 7 (100%) | |
| Total | 9 (100%) | 1 (11%) | 8 (89%) | |
| 1 McNemar’s test | | | | |

## Slide 7
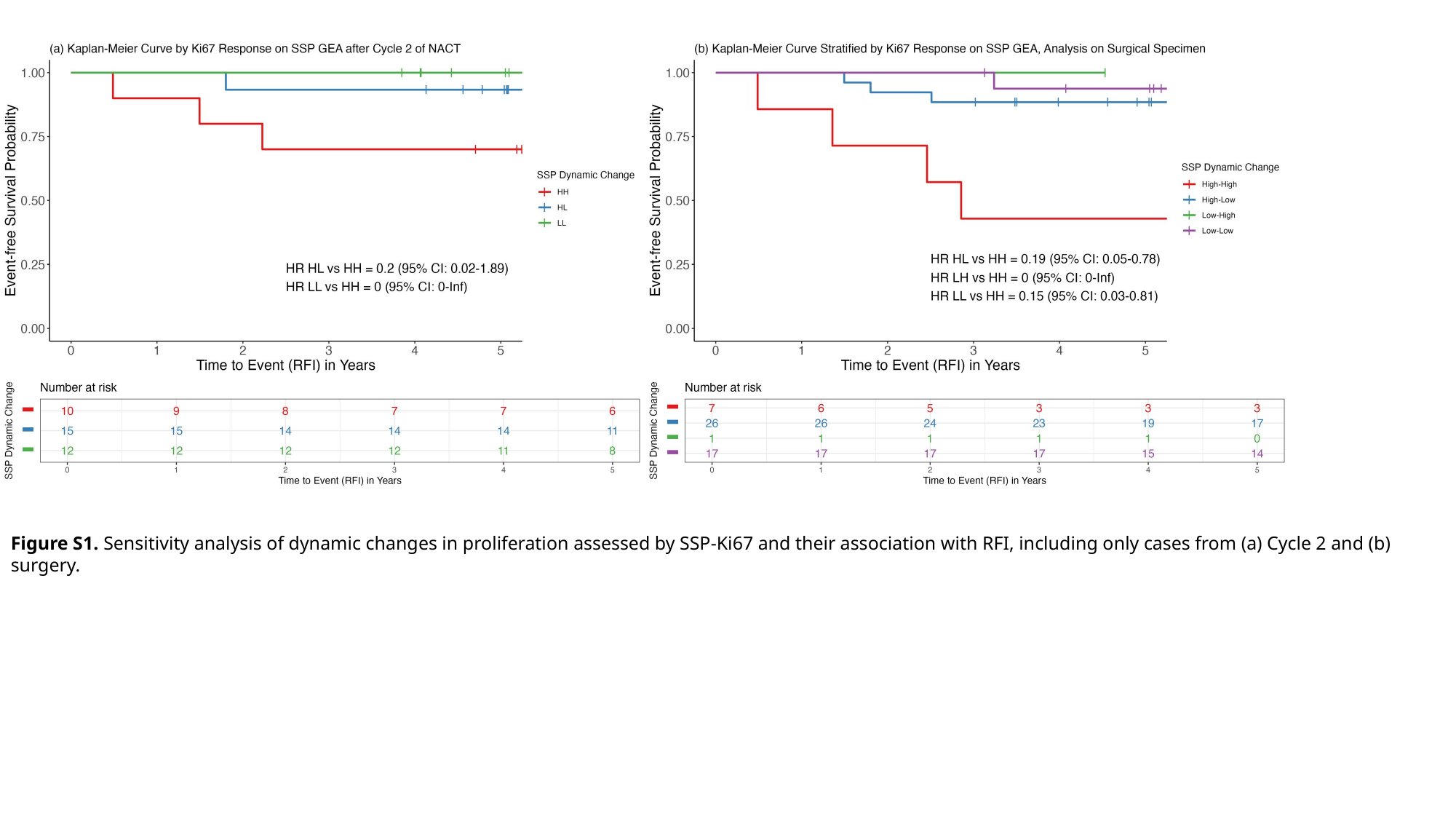

Figure S1. Sensitivity analysis of dynamic changes in proliferation assessed by SSP-Ki67 and their association with RFI, including only cases from (a) Cycle 2 and (b) surgery.
